# Impact of changes in perceived attentional function on postsurgical health-related quality of life in breast cancer patients awaiting adjuvant treatment

**DOI:** 10.1186/s12955-020-01485-y

**Published:** 2020-07-14

**Authors:** Mi Sook Jung, Moira A. Visovatti, Eun Hee Sohn, Hwa-Seung Yoo, Mijung Kim, Je Ryong Kim, Jin Sun Lee

**Affiliations:** 1 College of Nursing, Chungnam National University, 266 Minhwa-ro, Jung-gu, Daejeon, 35015 South Korea; 2 School of Nursing, University of Michigan, 400 N. Ingalls St, Ann Arbor, MI 48109 USA; 3 Department of Neurology, Chungnam National University Hospital, 33 Munwha-ro, Jung-gu, Daejoen, South Korea; 4 East-West Cancer Center, Daejeon University Korean Medicine Hospital, 176 Bun-gil, Daedeok-ro, Seo-gu, Daejeon, South Korea; 5 Department of Surgery and Research Institute for Medicinal Sciences, College of Medicine, Chungnam National University, 33 Munwha-ro, Jung-gu, Daejoen, South Korea

**Keywords:** Attention, Quality of life, Lymphedema, Breast neoplasms

## Abstract

**Purpose:**

Few studies have assessed pre-surgery cognitive impairment or the impact of pre-surgery cognitive impairment on quality of life. The purpose of this study was to assess changes in perceived cognitive function from pre-surgery to 1 month post-surgery and to determine whether cognitive function predicted health-related quality of life in women who awaited adjuvant treatment for breast cancer.

**Methods:**

This study used a descriptive pre-post design to assess women newly diagnosed with breast cancer prior to any treatment (*N* = 132). Cognition was assessed using the Attentional Function Index (AFI) and health-related quality of life was assessed using the Functional Assessment of Cancer Therapy-General (FACT-G). Statistical methods included descriptive, comparative and regression analyses. Covariates assessed and controlled for in analyses included depressed mood, fatigue, disturbed sleep, surgery-related symptoms (lymphedema/decreased mobility), and cultural tendency.

**Results:**

Perceived attention and memory function decreased from pre-surgery to 1 month post-surgery alongside alterations in arm function and a decrease in depressed mood (*p* < 0.05). Regression analysis indicated that, after controlling for covariates, poorer perceived attention and memory function, surgery-specific symptoms, and a greater tendency toward collectivism predicted poorer quality of life.

**Conclusion:**

Perceived function on tasks requiring attention and working memory 1 month post-surgery was poorer compared to pre-surgery suggesting that the mental and physical demands of a new diagnosis of breast cancer and surgery may effect cognitive function. Additionally, changes in perceived cognitive function significantly predicted perceived quality of life in women awaiting adjuvant treatment for breast cancer. Findings suggest that breast cancer patients are at risk for an early decline in cognitive function and that interventions aimed at supporting and optimizing function may improve quality of life early in the disease trajectory.

## Background

Women with breast cancer can experience cognitive symptoms across the disease trajectory from diagnosis to 20 years post-treatment [1]. In particular, cognitive dysfunction has been observed in approximately 30% of breast cancer patients prior to any treatment [2]. Longitudinal studies assessing subjective cognitive complaints in women with breast cancer have generally examined cognitive function from a post-surgery baseline [3, 4]. Findings from these studies suggest a pattern of decline and stabilization/recovery with perceived cognitive problems greatest after chemotherapy. However, one longitudinal study by Chen and colleagues [5] assessed perceived attention and working memory function from pre-surgery to 2 years after surgery. Findings showed that perceived problems with daily tasks requiring attention and memory were greatest 1 month after surgery followed by a gradual improvement over the remaining assessment period. This pattern of perceived decline and recovery in attention and working memory function is consistent with other studies using objective measures of function [6, 7]. Together, the review of the literature suggests the need for further research to assess perceived attention and working memory function during the surgical period when cognitive decline may occur even before chemotherapy initiation.

Cognitive decline may interfere with physical, mental, emotional, and social wellbeing or health-related quality of life in individuals with cancer [8, 9]. In breast cancer, poorer perceived cognitive functioning has been associated with psychological and physical distress [9, 10]. In particular, Von Ah and colleagues [9] assessed the relationship between perceived attention and working memory function and psychological and physical wellbeing in 134 breast cancer survivors approximately 6 years after diagnosis. Findings showed that lower perceived effectiveness on everyday tasks requiring attention and working memory predicted depressive symptoms, fatigue, poorer physical functioning, and overall wellbeing.

Theoretically, attention and working memory processes are necessary for goal-directed behavior, problem solving, and social functioning [11]. Alterations in these cognitive domains can reduce an individual’s personal effectiveness and ability to interact in a purposeful way [12]. To our knowledge, the relationship between perceived attention and memory function and health-related quality of life has not been assessed in breast cancer patients early in the disease trajectory from pre-surgery to 1 month post-surgery. Understanding this relationship has important implications for developing clinical strategies to reduce feelings of distress, optimize function, and improve health-related quality of life in individuals with breast cancer. Thus, the purpose of the current study was to assess changes in perceived attention and memory function in the early phase of treatment from diagnosis to post-surgical period after completing the first and only treatment and to examine the effect of cognitive change on post-surgery health-related quality of life controlling for possible covariates in women awaiting adjuvant treatment for newly diagnosed non-metastatic breast cancer.

## Methods

### Study design

This prospective descriptive study examined cognitive changes and the impact of cognitive changes on health-related quality of life in women who underwent surgery for breast cancer between February and September in 2016. Baseline assessment occurred within a week before any planned surgery (T1) and the second assessment occurred within a month following baseline assessment, coinciding with about 2 weeks after surgery and before any adjuvant treatment (T2). This study was approved by the Institutional Review Board of the Chungnam National University Hospital.

### Participants and setting

One hundred and thirty-two women newly diagnosed with localized breast cancer were recruited from the Cancer Center Breast Clinic of Chungnam National University Hospital located in a central region of South Korea. This hospital is a referral center for cancer patients across the central region and approximately 300 women have received surgical treatment for their newly diagnosed breast cancer at this center every year. All participants were female, at least 18 years old, able to read and speak Korean, and scored at least 3 on the Mini-Cog™ to ensure the absence of severe cognitive disorders such as dementia [13]. Exclusion criteria included: the presence of metastatic cancer, clinical depression, secondary diagnosis of neurological or psychiatric disorders, and coexisting debilitating medical conditions that could affect abilities to answer questionnaires.

Sample size was calculated by power analysis based on findings from previous studies assessing cognitive function and quality of life of cancer survivors [8, 14] with G power program version 3.1. A sample of 108 provided 85% power to detect small to medium effect sizes with 12 predictors at the 5% level of significance using multiple regression analysis. As such, a sample of 132 was found to be sufficient to obtain a statistical variance appropriate for the purpose of the current study.

### Study procedure

Women were identified through the hospital patient registration system. Data related to cancer and surgery were obtained and used to select patients who met the inclusion criteria of the study. When the selected women visited a clinic within a week before surgery, they were asked to participate in this study. Among 202 women who were asked to participate, 140 women enrolled. For the 62 women who declined, reasons included either too busy/not enough time to participate or their family caregivers refused to participate in the study. At the second assessment, 8 women were excluded because of physical weakness (*n* = 3) or withdrew secondary to busy schedules (*n* = 5).

Informed consent was obtained from all participants before the baseline assessment (Time 1). The second assessment occurred at a routinely scheduled follow up appointment in clinic (Time 2). Cognitive symptoms alongside demographic and medical characteristics, cultural characteristics, and other commonly occurring breast cancer symptoms were measured at Time 1. All physical, psychological, and cognitive symptoms which were assessed at Time 1 and health-related quality of life were measured at Time 2. All interviews were conducted after patients reported an absence of any surgical discomfort. The mean interval between Time 1 and Time 2 was 15.14 days (range: 8–34 days).

### Measures

#### Attentional Function Index

The Attentional Function Index (AFI) is a 16-item measure developed to assess an individuals’ perceived effectiveness in performing daily activities requiring attention, working memory, and executive function [12]. Each item is scored on a 11-point Likert scale ranging from 0 (not at all) to 10 (extremely well or a great deal). Higher scores indicate better cognitive functioning. The AFI is a valid and reliable instrument in individuals with cancer [12, 15]. The Cronbach’s alpha coefficients for Time 1 and Time 2 were .88 and .92, respectively, indicating satisfactory reliability.

#### Patient Health Questionnaire

The Patient Health Questionnaire (PHQ) is a self-report measure that assesses depressed mood in individuals [16]. The PHQ consists of 8 items with each item scored on a 4-point scale ranging from 0 (not at all) to 3 (nearly every day). Higher scores indicate worse depressed mood. The Cronbach’s alpha coefficients for Time 1 and Time 2 were .80 and .81, respectively, indicating good reliability.

#### Pittsburgh Sleep Quality Index

The Pittsburgh Sleep Quality Index (PSQI) is a self-report measure that assesses seven domains of sleep namely quality, latency, duration, habitual efficiency, disturbance, use of sleeping medications, and daytime dysfunction [17]. The total score was calculated by adding means on each PSQI domain. Higher scores indicate poorer quality of sleep. The Cronbach’s alpha coefficients for Time 1 and Time 2 of the current study were .72 and .73, respectively, indicating acceptable reliability.

#### Breast Cancer Prevention Trial Symptom Checklist

The Breast Cancer Prevention Trial (BCPT) Symptom Checklist was developed to assess commonly reported symptoms in breast cancer patients including vasomotor symptoms, urinary symptoms, vaginal symptoms, body image and weight problems, cognitive problems, and arm problems (lymphedema/ reduced mobility) [18]. In this study two items were selected from the 18-item BCPT symptom checklist and administered to assess surgery-related symptoms, namely arm lymphedema and mobility. The instrument asks each respondent to rate how bothersome a symptom is ranging from 0 (not at all) to 4 (extremely). The BCPT Symptom Checklist is a valid and reliable measure in women with or at risk for breast cancer [18].

#### Auckland Individualism and Collectivism Scale

The Auckland Individualism and Collectivism Scale is a self-report measure developed to assess critical attributes of collectivism (11 items) and individualism (15 items), respectively [19]. Respondents are asked to indicate the frequency of their attitudes, values, and beliefs using a 6-point Likert scale ranging from 1 (never or almost never) to 6 (always). In this study, the Cronbach’s alpha coefficient for Time 1 was .77, indicating acceptable reliability.

#### Functional Assessment of Cancer Therapy

We used the Functional Assessment of Chronic Illness Cancer Therapy-Fatigue (FACT-F) and the Functional Assessment of Chronic Illness Cancer Therapy-General (FACT-G). The FACIT-F is a self-report measure developed to assess current level of fatigue and the impact of fatigue on everyday functioning in patients receiving treatment for a variety of cancer diagnosis [20]. The FACIT-F consists of 13 items with each item scored on a 5-point Likert scale ranging from 0 (not at all) to 4 (very much so). Higher scores indicate greater fatigue within the past week. For this study, the Cronbach’s alpha coefficients of this measure for Time 1 and Time 2 were .92 and .94, respectively, indicating excellent reliability. The FACT-G which was a self-reported outcome measure was used to assess health-related quality of life in women undergoing surgery for localized breast cancer [21]. The Korean version of the FACT-G is composed of four domains including physical, social/family, emotional, and functional well-being. Respondents rate their level of health-related quality of life using 5-point Likert scales from 0 (not at all) to 4 (very much). Higher scores indicate better quality of life. For this study, Cronbach’s alpha coefficient at Time 2 was .89, indicating good reliability.

### Statistical analysis

Statistical analyses were performed using IBM SPSS version 24.0 for Windows. Descriptive statistics were used to analyze demographic and medical characteristics, physical, psychological, and cognitive problems, cultural tendency toward collectivism, and health-related quality of life. Comparative analyses with paired t-tests were performed to determine whether there were statistically significant changes in cognitive function, depression, fatigue, sleep problems, and surgery-related arm problems. Multiple regression analyses were performed to assess the effect of changes in cognitive function on health-related quality of life, when controlling for changes in co-occurring symptoms including depression, fatigue, sleep problems, and surgery-related arm problem, cultural tendency, and demographic and medical variables selected from results of post-hoc analyses in this study.

## Results

### Sample characteristics

Sample characteristics were presented in Table 1. Most participants were middle-aged (M = 50.80, SD = 9.96), well-educated (M = 12.24, SD = 3.24), married (*n* = 105, 79.5%), and unemployed (*n* = 81, 61.4%). For medical characteristics, more than half of participants were premenopausal (*n* = 71, 53.8%) and did not have comorbid health problems (*n* = 75, 56.8%) before surgery. All women were newly diagnosed with localized breast cancer (stage 0-III) and received lumpectomy (43.2%) or mastectomy (56.8%).

**Table 1:** Sample characteristics

		M (SD)	n (%)
Age (years)		50.80 (9.96)	
Education (years)		12.24 (3.24)	
Time interval between T1 and T2 (days)		15.14 (4.28)	
Histological type	DCIS		33 (25.0)
	Ductal		83 (62.9)
	Lobular		9 (6.8)
	Others		5 (3.8)
	Unknown		2 (1.5)
Stage of cancer	0		34 (25.8)
	I		51 (38.6)
	II		35 (26.5)
	III		12 (9.1)
Surgery	Lumpectomy		57 (43.2)
	Mastectomy		75 (56.8)
Axillary surgery	Sentinel node biopsy		96 (72.7)
	Axillary dissection		34 (25.8)
	Unknown		2 (1.5)
Comorbidity	Yes		57 (43.2)
	No		75 (56.8)
Menopausal status	Premenopausal		71 (53.8)
	Perimenopausal		5 (3.8)
	Postmenopausal		56 (42.4)

### Physical, psychological, and cognitive problems before and after surgery

**Fig. 1:**
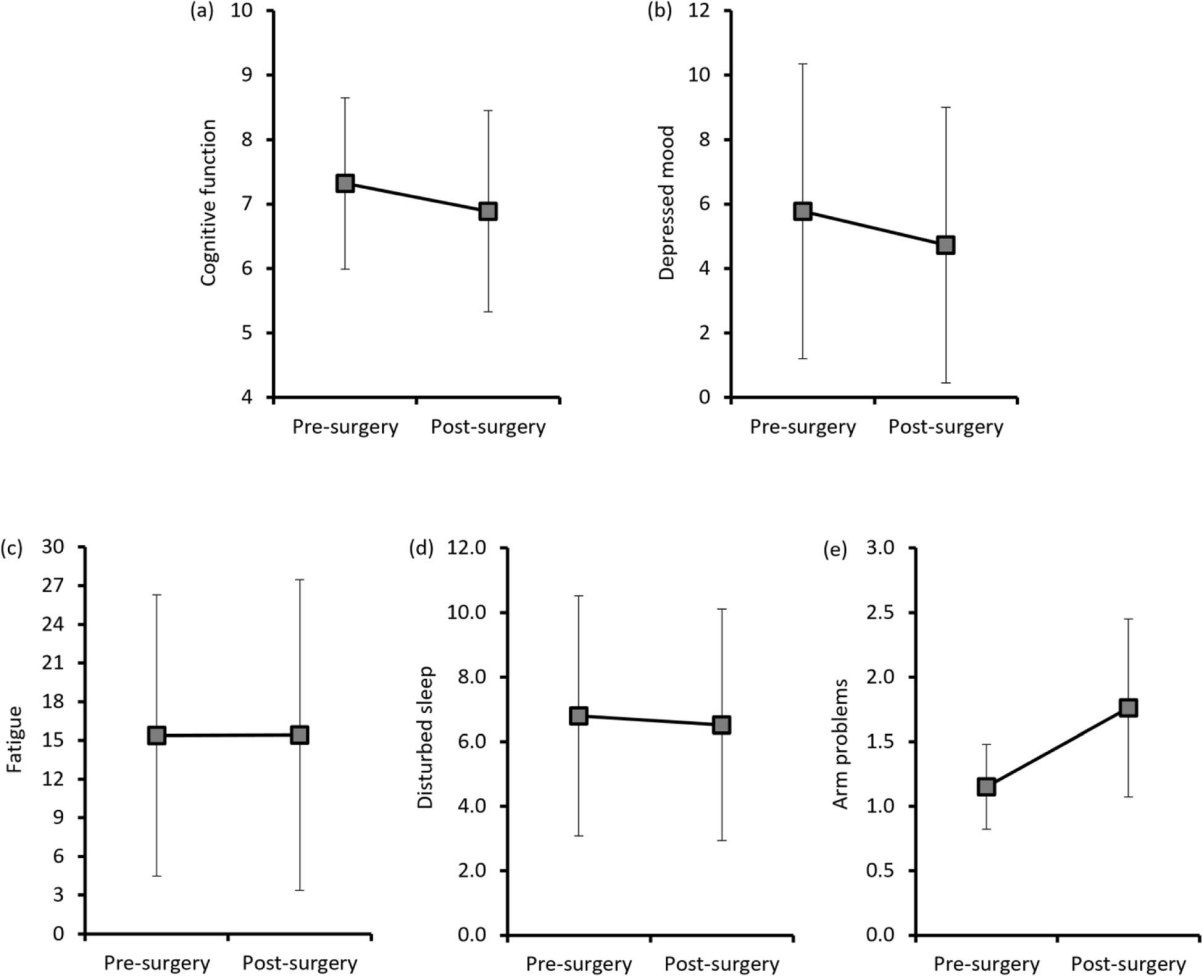
Changing patterns of symptom distress variables by group before and after surgery. **a** Perceived cognitive function assessed with AFI (lower scores = more complaints), **b** Depressed mood assessed with PHQ (higher scores = greater severity), **c** Fatigue assessed with FACT-F (higher scores = greater severity), **d** disturbed sleep assessed with PSQI (higher scores = greater severity), **e** Arm problems assessed with two items of BCPT symptom checklist (higher scores = greater severity)

As presented in Fig. 1, paired t-test analyses indicated statistically significant changes in cognitive function (t = 3.40, *p* < .001), depressed mood (t = 2.99, *p* = .003) and arm function (t = − 10.47, *p* < .001) from before to after surgery. This finding indicated that alterations in cognition and arm function after surgery was greater than those before surgery while depressed mood improved over time. Based on the psychometric properties of the PHQ, a cut-off score of ≥10 is considered suggestive of clinical depression [16]. Nineteen percent of the participants before surgery and 12% of participants after surgery had PHQ scores suggestive of clinical depression. No significant changes in fatigue and sleep problems were found between two time points. However, the occurrence of these symptoms somewhat differed in both time points. While less than 10% of participants reported clinically significant fatigue [22] before and after surgery (4.5% at Time 1; 9.1% at Time2), more than 40% of them had clinically meaningful sleep problems [18] in both time points (46.1% at Time 1; 43.8% at Time 2).

### Correlations among factors associated with health-related quality of life

**Fig. 2:**
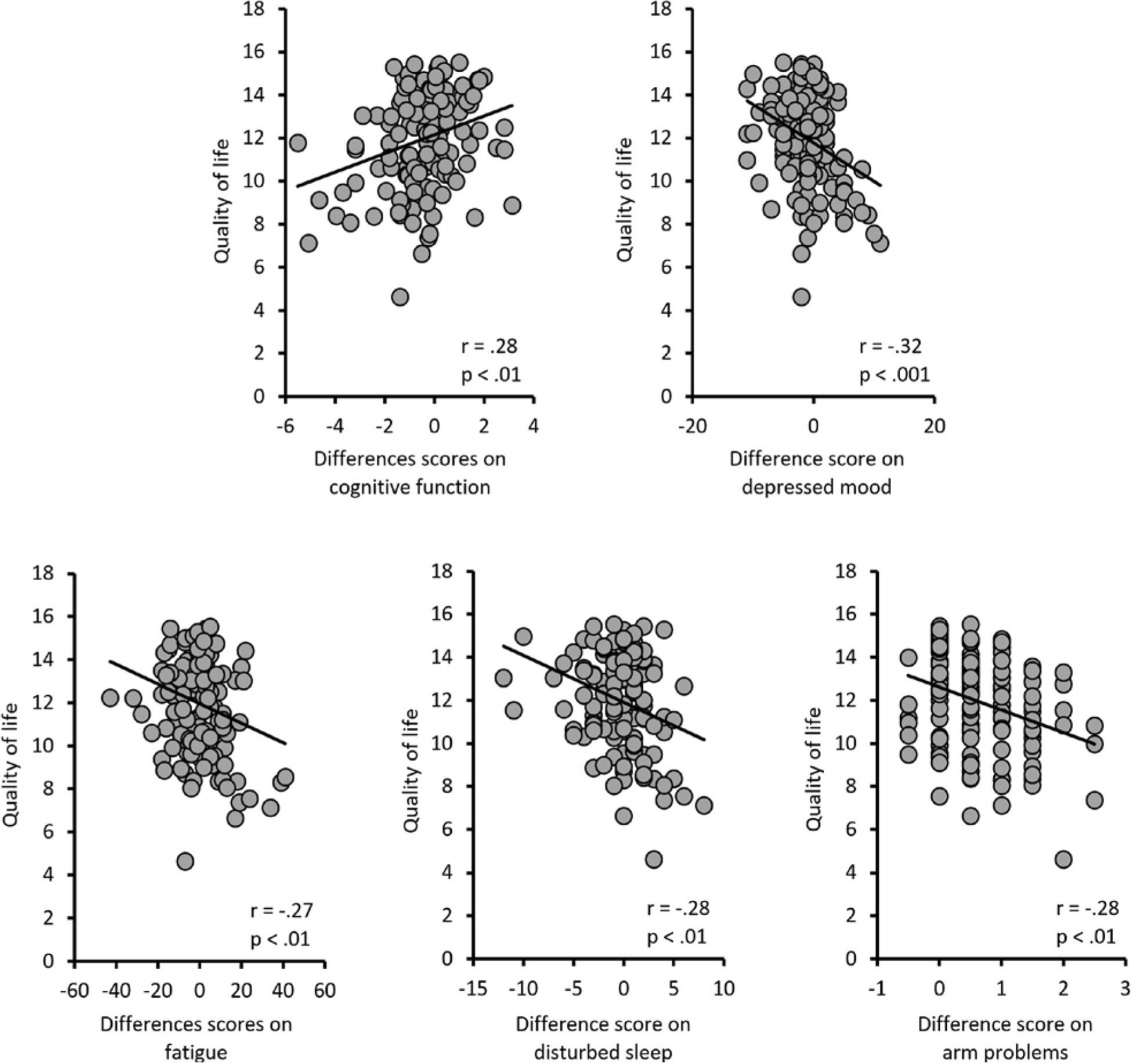
Correlations of health-related quality of life (x-axis) and difference scores on symptom distress variables

Pearson correlation coefficients were computed to assess the relationships between post-surgery health-related quality of life and changes in cognitive function, depressed mood, fatigue, sleep problems, arm function as well as baseline cultural characteristics and selected demographic characteristics. As seen in Fig. 2, health-related quality of life was associated with symptom distress. Specifically, lower health-related quality of life was associated with lower perceived effectiveness on daily tasks requiring attention and memory function (*r* = .28, *p* = .001), worse depressed mood (*r* = −.32, *p* < .001), greater fatigue (*r* = −.27, *p* = .002), poorer quality of sleep (*r* = −.28, *p* = .001), and surgery-related arm problems (*r* = −.28, *p* = .001). Also, health-related quality of life was correlated with collectivism (*r* = .23, *p* = .009) and individualism (*r* = .28, *p* = .001) but not demographic characteristics such as age or educational level.

### Predictors of health-related quality of life

Multiple regression analysis was performed to determine the impact of changes in cognitive function on health-related quality of life early in the breast cancer disease trajectory, controlling for potential covariates including symptom distress (depressed mood, fatigue, altered arm function, sleep problems) age, education, type of surgery (lumpectomy, mastectomy), stage of illness (stage 0-III), and cultural characteristics (collectivism, individualism). The regression model was significant (Adjusted *R*^*2*^ = .298, F (12,120) = 5.68, *p* < .001), with a review of betas revealing that changes in cognitive function (β = .23, *p* = .006) and arm function (β = −.30, *p* < .001) and a tendency toward collectivism (β = .19, *p* = .018) were associated with health-related quality of life after surgery prior to any adjuvant treatment (Table 2). Findings indicate that increases in cognitive and physical symptoms (altered arm function) were associated with lower health-related quality. Greater tendency toward collectivism was a significant factor of better health-related quality of life but not a tendency toward concurrent individualism and health-related quality of life.

**Table 2:** Factors associated with health-related quality of life after surgery for breast cancer

Variables	B	SE	*p*
Age	.18	.03	.102
Education	.08	.07	.433
Stage of cancer	.06	.18	.441
Type of surgery (1 = mastectomy)	.12	.35	.117
Comorbidity (1 = having comorbid condition)	−.10	.39	.272
Collectivism	.19	.25	.018
Individualism	.14	.28	.089
Difference score on cognitive function	.23	.13	.006
Difference score on depressed mood	−.09	.05	.344
Difference score on fatigue	−.11	.02	.200
Difference score on disturbed sleep	−.14	.07	.125
Difference score on surgery-related arm problems	−.30	.26	<.001

## Discussion

The current study examined cognitive symptoms in individuals with localized breast cancer before and after surgery and the impact of cognitive symptoms on health-related quality of life to inform the development of early interventions to maintain and/or optimize function, improve quality of life, and support the survival of breast cancer survivors.

Study findings indicated that individuals with breast cancer perceived worse effectiveness on daily tasks requiring attention and memory alongside changes in arm function and improved depressed mood from diagnosis to post-surgery. Findings of worse cognitive problems post-surgery are consistent with previous studies in women with breast cancer [5, 7]. Specifically, Chen and colleagues [5] found that women diagnosed with non-metastatic breast cancer reported lower scores on perceived effectiveness on daily tasks requiring attention and memory (AFI = 6.7) 1 month after surgery compared to pre-surgery (AFI = 8.17) and gradual recovery over 2 years toward the baseline pre-surgical assessment. Additionally, Cimprich and Ronis [7] assessed attention and memory function using a brief battery of neuropsychological tests, symptom distress using a questionnaire with 10 common symptoms including changes in mobility, and depressed mood in women with breast cancer before surgery, 2 weeks post-surgery and 3 months post-surgery. Findings from repeated measures ANOVA indicated significant gain in attention and working memory function from pre-surgery to 3-months post-surgery but not 2 weeks, no significant changes in symptom distress at 2-weeks or 3-months and a significant improvement in depressed mood from pre-surgery to 2-weeks post-surgery. For symptom distress, the instrument chosen did not include a specific item on arm mobility or lymphedema and thus may not have fully captured distress related to function. Together findings suggest that changes in cognitive function may have occurred secondary to physical stress from the surgical intervention and/or mental fatigue from the cognitive demands associated with coping with new diagnoses of cancer and adjusting to functional changes associated with breast cancer surgery [7, 23]. To our knowledge, the current study is the first report to provide evidence for changes in perceived cognitive function during a critical period prior to adjuvant treatment in ethno-culturally homogenous Asian women with non-metastatic breast cancer.

Multiple regression analyses found that, after controlling for possible covariates, a change in perceived cognitive function from pre- to post-surgery was found to significantly predict health-related quality of life after surgery. Findings are congruent with Von Ah and colleagues study assessing perceived attention and memory and quality of life in women approximately 6 years since diagnosis [9] as well as Hutchinson and colleagues observation of correlations between perceived cognitive impairment and emotional distress [10]. In particular, findings from Von Ah and colleagues’ study indicated that scores on the AFI predicted psychological and physical wellbeing after controlling for age, education, race and comorbid conditions [9]. Together, findings suggest that interventions aimed at maintaining and/or optimizing cognitive function at diagnosis and early in the disease trajectory may support post-treatment health-related quality of life. Non-pharmacological interventions that hold promise include: Cognitive training, physical activity, Tibetan sound meditation, mindfulness-based music therapy, natural environment or restorative therapy, neurofeedback, yoga, psychoeducation, and cognitive behavioural therapy [24]. These interventions may exert an effect through a variety of mechanisms including immune regulation, neurologic modification, stress reduction, cognitive training, and restoring attentional function [24].

Alterations in arm function was also found to be a significant predictor of post-surgery health-related quality of life in women newly diagnosed with breast cancer but not depressed mood, fatigue or sleep problems. Alterations in arm function, such as reduced range of motion and lymphedema, got worse over time although physical condition was recovered without serious levels of complications from surgery. The finding that changes in arm function (lymphedema/decreased mobility) was associated with health-related quality of life is consistent with a previous study showing arm functions were found to be a significant predictor of reduced quality of life in 990 breast cancer patients [25].

In the current study a greater tendency toward collectivism was found to be associated with better health-related quality of life, suggesting that preserving some characteristics of a collectivistic culture may support quality of life in women with breast cancer. This finding supports a theoretical construct that an individuals’ quality of life is likely to be modulated by cognitive appraisal made in the cultural contexts in which they have lived in relations to their values, expectations, and satisfaction [26]. From the perspective of cultural psychology, individuals from collectivist cultures are more likely to consider negative emotional responses to unpleasant situations as a normal, inevitable, or acceptable state. In contrast, individuals from individualist cultures are more likely to consider negative emotional responses to unpleasant situations as a problem that needs to be fixed [27]. Given that individuals from collective cultures tend to accept negatives as part of their lives, women raised in this culture may find cognitive solutions to evaluate their stressful situations as tolerable, possibly leading to a better quality of life. However, this is not the case for Asian women who tend to distance themselves from an “over-negative” state, leading to a decreased ability to accepting their condition, communicate changes in wellbeing with healthcare providers, and seek support [28]. Thus, future studies should identify which culture-specific factors can make breast cancer patients less inclined to express their distress related to cancer management. Additionally healthcare professionals need to assess health-related quality of life differently depending on the cultural contexts.

Changes in depressed mood did not significantly predict health-related quality of life in this study. This was an unexpected finding as previous research suggests that depressed mood can diminish health-related quality of life by negatively affecting physical functioning, vocational performance, interpersonal relationships, and even survival in breast cancer patients [29]. However, Cimprich and Ronis [7] also found that psychological distress improved from pre to post surgery. One explanation for this finding is that participants’ self-report of depressed mood early in the disease trajectory was in the low to moderate range and low levels of distress were not enough to affect health-related quality of life. Alternatively, depressed mood may have dissipated after initial feelings of shock, disbelief and fear related to a new diagnosis of cancer [30]. Given these inconsistencies, additional research is needed to assess psychological distress at diagnosis and during the initial surgical period.

Fatigue and sleep problems did not significantly predict health-related quality of life in this study. One explanation for this finding is that changes in fatigue and sleep problems may not have been great enough to affect health-related quality of life. Interestingly, the number of women reporting a clinically significant level of fatigue was small before and after surgery while roughly half of women had sleep problem regardless of whether they were in pre-surgical or post-surgical period. Thus, it is suggested that future research carry out comprehensive physical, psychological, and cognitive assessment from the pre-surgical period to detect post-surgery quality of life impairment in early stage.

There are several limitations of our study. First, this study did not include objective neuropsychological parameters. Although we used the well-developed self-report instrument, the instrument may not capture cognitive changes of a subset of individuals who demonstrated changes in neuropsychological performance but not self-report. Second, the study sample consisted of an ethnically and culturally homogeneous group of women with breast cancer and did not have culture-matched comparison group of breast cancer patients from an individualistic society. Despite these limitations, this study provides new evidence of changes in cognitive function on health-related quality of life and suggests the need to assess health-related quality of life within a cultural context.

## Conclusion

Perceived cognitive function declined from pre- to post-surgery in women newly diagnosed with breast cancer. Importantly, cognitive decline was associated with lower health-related quality of life regardless of stage of illness or extent of surgery. Findings suggest the need for interventions to support cognitive function early in the breast cancer disease trajectory to optimize function and improve health-related quality of life.

## Data Availability

The datasets generalized and/or analyzed during the present study are available from the corresponding author on reasonable request.

## Abbreviations

AFI: Attentional Function Index
PHQ: Patient Health Questionnaire
FACT-F: Functional Assessment of Cancer Therapy-Fatigue
PSQI: Pittsburgh Sleep Quality Index
BCPT: Breast Cancer Prevention Trial
FACT-G: Functional Assessment of Cancer Therapy-General
